# Ipsilateral ectopic pregnancy occurring in the stump of a previous ectopic site: a case report

**DOI:** 10.1186/1757-1626-1-343

**Published:** 2008-11-21

**Authors:** Bode-Law Faleyimu, Gabriel O Igberase, Mojeed O Momoh

**Affiliations:** 1 Chevron Hospital Warri, Delta State, Nigeria; 2 Department of Obstetrics and Gynaecology, College of Health Sciences, Delta State University, Abraka, Delta state, Nigeria; 3 Irrua Specialist teaching hospital, Irrua, Edo State, Nigeria

## Abstract

**Background:**

Ectopic pregnancy continues to be a significant cause of maternal morbidity, mortality and reproductive failure in Nigeria. Ipsilateral ectopic pregnancy occurs rarely and may be difficult to diagnose in low resource settings where there are no diagnostic tools. Few cases have been reported in the literature but none in our region.

**Case presentation:**

We present an unusual case of a 22 year old female undergraduate, from the Urhobo tribe in the Niger Delta region of Nigeria who had a recurrent left ectopic pregnancy at the stump of a previous cornual resection done five years earlier. She had a left salpingo-oophorectomy done and did well postoperatively.

**Conclusion:**

Ectopic pregnancy could pose a diagnostic dilemma where diagnostic facilities are not available. Every woman with a previous ectopic pregnancy would be at high risk for recurrence and that would be the condition to be ruled out if a pregnant woman presented at early gestation with abdominal pain.

## Background

Ectopic pregnancy continues to be an important contributor to maternal mortality, morbidity and early fetal wastages in the first trimester of pregnancy [1]. The incidence of recurrent ectopic pregnancy is approximately 15% and this rises to 30% following two ectopic pregnancies [2]. A previous study on ectopic pregnancy done in the same region revealed an incidence of 3.5% of total hospital births [1].

Management of ectopic pregnancy has been improved upon by the use of ultrasound, laparoscopy and monitoring of beta subunit of human chorionic gonadtrophin[3]. In developed nations, treatment options have shifted from laparotomy to conservative surgical and non surgical techniques.

Ectopic pregnancy could pose a diagnostic dilemma in centres where diagnostic facilities are lacking. We present an unusual case of left ipsilateral ectopic pregnancy occurring in the stump of a previous ectopic site following cornual resection.

## Case presentation

We present the case of a 22 year old Para 0^+2 ^female undergraduate from the Urhobo tribe in the Niger Delta region of Nigeria who was seen at the gynecological clinic with a two week history of colicky lower abdominal pain which became generalized a day prior to presentation. There was no history of dizziness or fainting spells. There were no gastrointestinal symptoms. She bled per vaginam two weeks prior to presentation for four days which she claimed to be her menses. There was no history of chronic cough or weight loss. She did not have any purulent vaginal discharge prior to bleeding per vagina.

She had a previous left cornual resection for a ruptured ectopic pregnancy five years ago in this hospital. There was also a history of having been managed for sepsis following the termination of an eight week pregnancy four months prior to presentation.

Examination revealed that she was in painful distress and had mild pallor. Chest was clinically clear. Her pulse rate was 100 beats per minute while the blood pressure was 120/70 mmHg. Abdomen examination revealed a full, soft abdomen with tenderness in the suprapubic region, right and left iliac fossae, left renal angle and periumbilical area. Vaginal examination showed a normal lower genital tract, uterus was normal sized, anteverted, no pelvic masses were felt, pouch of Douglas was empty and there was marked cervical excitation tenderness. Differential diagnoses were those of left pyelonephritis, pelvic inflammatory disease and slow leaking ectopic pregnancy. Complete blood count and urinalysis was normal. Hemoglobin was 10.6 gm/dl. Serum beta human chorionic gonadotrophin was 3,500 mIU/mL. Screening for chlamydia and gonorrhoea were negative. Pelvic ultrasound scan revealed the presence of a left adnexal complex mass with associated free pelvic fluid and increased blood flow activity. The uterus shows a normal outline with slightly thickened endometrial plate and no intrauterine cyesis. Conclusion was left ectopic pregnancy. She was placed on a nil per os regimen and intravenous fluid was commenced. Two units of whole blood were grouped and cross-matched.

A left salpingo-oophorectomy with pelvic adhesiolysis was done. Repeat Pfannenstiel incision through previous scar was made to enter the abdominal cavity. Findings at surgery were hemoperitoneum of 600 mls, left tubo-ovarian mass adherent to the pelvic floor (left chronic ectopic involving the tubal stump and the ovary with vesico-utero-ovarian adhesions), omento-uterine adhesions, cornual adhesions and stump, slightly bulky uterus, normal looking right ovary and a beaded right fallopian tube.

The haemoperitoneum was sucked and adhesiolysis performed to free the omentum from behind the uterus. The left tubo-ovarian mass was lifted out and bluntly freed from the pelvic floor to define a near anatomy. A left salpingo-oophorectomy was performed and haemostasis was secured. Abdominal wall was closed with subcuticular dexon to skin. She was discharged on the fourth postoperative day.

She was counseled on the use of contraception and the combined oral contraceptive was offered her. The possibility of repeat ectopic pregnancy was explained and she was counseled on safe sex. She was seen again two weeks later and was found to be stable.

Histology report revealed a left tubal ectopic pregnancy and a left ovarian corpus luteum cyst. There was no evidence of genital tuberculosis.

## Discussion

She had a previous cornual resection done and she still had a repeat episode on the stump of the same fallopian tube. There was no history of this patient being on any contraceptive method. There was also a positive history of septic abortion which is a risk factor for ectopic pregnancy. This, in combination with a history of previous ectopic, which is the most significant risk factor, favoured the setting for a repeat ectopic pregnancy [1,4]. The literature is replete with a lot of theories on the mechanism of recurrent ipsilateral ectopic pregnancy [5,6]. One theory explains that spermatozoa pass through the patent tube into the pouch of Douglas, then travel to fertilize the ovum on the side of the diseased tube. The fertilized ovum then implants on the stump of previous ectopic site. Another, is the theory of transperitoneal migration which says the fertilized ovum on the side of the normal tube migrates and gets implanted on the tubal stump. A third theory says despite ligation, lumina remain intact in the interstitial portion and distal remnant of the fallopian tube. This allows communication between the endometrial and peritoneal cavities and thus migration of the fertilized ovum or spermatozoa from the endometrial cavity to the distal remnant of fallopian tube.

**Figure 1:**
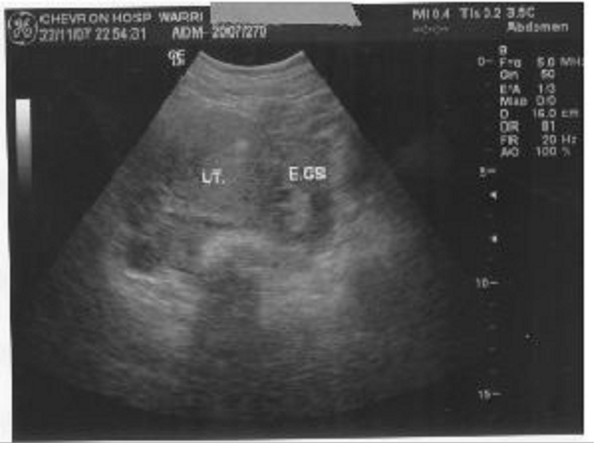
Showing the uterus (UT) and left tubal ectopic gestational sac (EGS).

Ectopic pregnancy has been described as the great masquerader. Figure 1 shows left tubal ectopic gestation. What looked like her last menstrual period lasted for four days. This would have confused the picture more. However, she had abdominal pain which is the commonest presenting symptom. Can we say this girl was lucky? In a way, yes. She had the ectopic pregnancy on the same fallopian tube previously affected. She still has more than 80% chance of intrauterine conception if the contralateral tube is healthy. Unfortunately it appeared beaded macroscopically. However if the only viable tube is affected, she will be left only with the choice of assisted conception. The patient was screened for Chlamydia, Gonorrhoea and genital tuberculosis. This rules out a pelvic inflammatory disease because of the possibility of recurrence and further complications. Tuberculosis is common in developing countries and with the presence of pelvic adhesions in this patient, is a good differential diagnosis. There was no history of chronic cough or weight loss. Genital tuberculosis was ruled out by histology.

Salpingo-oophoorectomy was done in this case as the fallopian tubes and ovaries were bound in adhesions.

Modern management involves the use of laparoscopy to perform salpingectomy and the conservative surgeries of salpingostomy and salpingotomy.

## Conclusion

Ectopic pregnancy could pose a diagnostic dilemma where diagnostic facilities are not available. Every woman with a previous ectopic pregnancy would be at high risk for recurrence and that would be the condition to be ruled out if a pregnant woman presented at early gestation with abdominal pain.

## Consent

Written informed consent was obtained from the patient for publication of this case presentation and accompanying image. A copy of the written consent is available for review by the editor-in-chief of this journal.

## Competing interests

The authors declare that they have no competing interests.

## Authors' contributions

BL did the recent surgery, provided data, read and approved the manuscript. GO assisted in the recent surgery, provided data, interpreted findings, read and approved final manuscript. MO performed the first surgery, provided data, interpreted and approved final manuscript.
